# Bringing fictional characters to life: reflections on co-creating a comic book with members of the public

**DOI:** 10.1186/s40900-023-00437-2

**Published:** 2023-05-02

**Authors:** Joanne Marie Cairns, Helen Roberts, Geraldine Al-Khafaji, Maria Kwater

**Affiliations:** 1 Hull York Medical School, University of Hull, Allam Medical Building, Cottingham, Hull, HU6 7RX UK; 2 Rye, East Sussex, UK; 3 Leeds, Yorkshire and the Humber, UK

**Keywords:** Public involvement, Bowel cancer, Screening, Comic, Visual elicitation, Co-creation

## Abstract

There are growing calls for cancer screening to become more personalised by considering a range of risk factors, rather than a one-size-fits-all, age-based approach. The aim of this public involvement was to co-create a comic book about bowel cancer screening to be used as a visual elicitation tool in research focus groups with members of the public and healthcare professionals, as part of the *At Risk* study, to discuss their attitudes toward personalised bowel cancer screening, which would involve considering different risk factors. This article critically reflects on the co-creation process to develop the comic book, benefits and challenges, and some lessons learned to inform other researchers considering a similar approach. In total, ten public contributors (5 men and 5 women) from two public involvement networks participated in two successive online workshops to develop six fictional characters, two for each level of bowel cancer risk (low, moderate and high risk). This tool was then used in the *At Risk* study comprising five focus groups involving 23 participants, including members of the public (n = 12) and healthcare professionals (n = 11). The co-created comic book was a generally well-received research tool able to generate discussion about a complex topic, bowel cancer risk, in an accessible way. It was suggested that the comic book may also be extended beyond the research context to inform bowel cancer screening decisions and raise awareness of risk factors. 
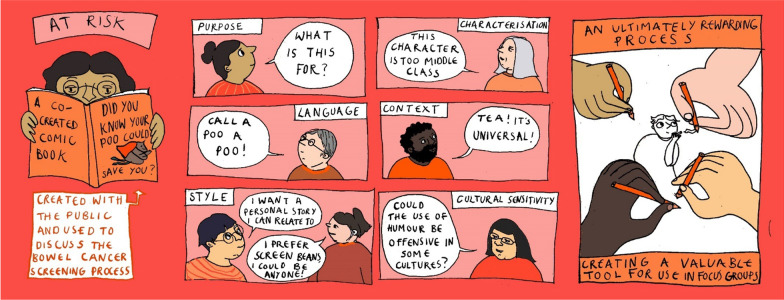

## Abstract (summary)

A national screening programme for bowel cancer was set up in 2006. This is offered every two years to people aged 60–74 in England and 50–74 in Scotland. Bowel cancer is the fourth most common cancer in the UK and the risk of developing bowel cancer increases with age. Smoking, alcohol, diet and lack of exercise also increase the risk. An individual can change their lifestyle to reduce their risk. But if someone has a history of bowel cancer in their family, or has had another type of cancer themselves, they are still more likely to develop bowel cancer. Certain ethnic groups are also at higher risk. There are calls to tailor the bowel cancer screening programme to each person’s level of risk. This needs to be acceptable to those who are offered screening. In our study, we spoke to members of the public and healthcare professionals to find out their views on this. This is a complex topic, and we decided to create a comic book to help people understand the issues and encourage discussion. We worked with an artist and ten public contributors to create the comic book. We held two online workshops and presented the comic book at a network for different ethnic groups in our region. Here we discuss what we learned from this creative process. We focus on: diversity in the workshops; the purpose of the comic book; language and humour; visual style; developing the characters; the setting for the story; cultural sensitivity and stigma.

## Background

Bowel cancer is the fourth most common cancer with almost 42,000 people diagnosed each year, equating to 1 in 15 men and 1 in 18 women [1]. The national Bowel Cancer Screening Programme (BCSP) was set up in 2006 with the aim of detecting bowel cancer at an earlier, more treatable, stage.

There are two stages to the BCSP: (1) a faecal immunochemical test (FIT) is offered every two years to people aged 60–74 years in England, although this is gradually being lowered to age 50 [2], and 50–74 years in Scotland; (2) if the FIT result is positive, a follow-up procedure called a colonoscopy is undertaken. Currently, the BCSP is based on age alone but there are many other risk factors for developing bowel cancer, including modifiable lifestyle factors (such as smoking, alcohol consumption, diet and lack of exercise) and non-modifiable factors (ethnicity, family history of bowel cancer and personal history of cancer) [3].

There have been growing calls in the UK, and internationally, for bowel cancer screening to move away from a one-size-fits-all approach to a more personalised approach. This would include consideration of the above risk factors to give a more precise estimate of a person’s risk and, therefore, tailor the screening they receive. In order to move in this direction, risk-based bowel cancer screening needs to be acceptable to its target population to avoid widening existing inequalities in uptake. The overall aim of the ‘*At Risk*’ study was to examine attitudes towards personalised bowel cancer screening among those eligible for screening and healthcare professionals alike.

The researchers chose a visual elicitation tool for the *At Risk* focus groups in order to communicate complex information, bowel cancer risk, in a clear and accessible way. In this study, we co-created a comic book with fictional characters to convey different levels of bowel cancer risk, drawing on modifiable and non-modifiable risk factors. The comic book was co-created with members of the public to ensure relevance and inclusivity, as well as recognising the importance of public involvement from the outset of a research project. Co-creation has been defined as a *creative* and *innovative* process, which *openly* and *actively* engages relevant stakeholders [4].

The use of visual elicitation in research is not new. Since around the 1980s, visual elicitation tools have been increasingly adopted by qualitative researchers as part of a wider creative movement to use more innovative methods, such as drawings, photographs and zines [5]. Visual methods belong to the family of participatory research, making them compatible with other qualitative methods, including focus groups and interviews, to build a richer picture [6].

Within health promotion research, comic books have been used to communicate educational messages in a visual way for younger or less literate audiences, with a view to encouraging people to change their behaviour [7], but their full potential in health settings has yet to be fully realised [8]. Although their use in cancer research is quite novel, there are examples where the comic book format has been used to promote healthy eating in the United States [9], to encourage cervical screening uptake in South Africa [10], and to communicate prostate cancer risk factors in Canada [11]. In all of these cases, comic books have helped to communicate complex information in an accessible and engaging way, using imagery to generate conversation and aid understanding [7]. The use of creative methods can also help to be more inclusive by involving often seldom heard voices into public involvement [12], thereby shaping the research from the outset.

Comic books can also be considered as a ‘third object prompt’ [13] in research, an approach traditionally utilised in social work research with children. Objects can act as a distraction from discussions around personal or emotional issues by enabling individuals to use an inanimate element to reflect, frame and attach their own meanings and understandings to objects and images [13]. They offer a critical distance for discussion on sensitive issues, re-directing the conversation away from the ‘personal’ and the ‘researcher’ to focus on what is presented in the comic book.

However, a systematic review [14], p.138] found that little is written about ‘*how* to incorporate the arts in health research and a paucity of critical debate’. In this article, we aim to address this through a critical reflection on co-creating a comic book with members of the public. It is hoped that by sharing some of the benefits and challenges, as well as some lessons learned, this will help other researchers who are considering adopting a similar approach.

## Our approach

The full protocol of the *At Risk* study is registered with the Open Science Framework [15]. In this section, our approach to the public involvement within this study is discussed. The objective of the public involvement was to co-create a comic book in order to illustrate different levels of bowel cancer risk, based on modifiable and non-modifiable risk factors, in a clear and accessible way. The comic book was used as a research tool during the focus groups to stimulate discussion about a complex topic sensitively.

We wanted to involve the public as active partners in our research, since the researchers value the experiential knowledge and expertise that public partners can bring. By public involvement, we refer to the following National Institute for Health and Care Research (NIHR) [16] definition:

…research being carried out ‘with’ or ‘by’ members of the public rather than ‘to’, ‘about’ or ‘for’ them. It is an active partnership between patients, carers and members of the public with researchers that influences and shapes research.

The NIHR has set out six standards for public involvement: (1) inclusive opportunities, (2) working together, (3) support and learning, (4) governance, (5) communication and (6) impact [17]. We used these standards as a guide in our study, with a particular focus on inclusivity.

### Recruitment of public contributors

The research team advertised the opportunity to get involved in co-creating the comic book through two public involvement networks, Involve Hull (based at the University of Hull) and Bowel Research UK’s People and Research Together. We received expressions of interest from 24 members of the public from the two networks combined. From those who came forward, we had funding to select ten people from across the UK who were eligible for bowel cancer screening, including people who had chosen not to participate in the screening programme. Two of these public contributors are co-authors on this article. Amongst the group, there was an even gender balance (5 men/5 women). The age range was 60 to 74 years. It was difficult to achieve ethnic diversity with the majority of those who volunteered being White British (91.7%). However, despite including all of those initial volunteers who were not White British, the group remained predominantly White British (8 out of 10). To address this imbalance, we also involved a local organisation, Humber All Nations Alliance, to make sure that what we were doing was culturally sensitive. Those who took part in our public involvement workshops were offered remuneration for their time and expertise as per the national guidance [17].

### Co-creation workshops

Two successive online workshops lasting 90 minutes each were conducted in Zoom in December 2021 and January 2022 with the same participants. The objectives of the workshops are outlined in Box 1.

**Box 1:** Workshop objectives

Workshop 1	Workshop 2
To learn more about the project and each other and share views on how we can recruit a diverse range of people to take part in public focus groups to discuss attitudes to personalised bowel cancer screening	To learn more about the fictional characters we wanted to develop and what we would like to achieve with these (accessible & engaging characters for discussion about bowel cancer risk in focus groups, avoiding stereotypes)

In advance of the first workshop, the research team emailed a two-page research summary outlining the overall aim of the project (written in Plain English) to public contributors, with an explanation of the scope of their involvement. All ten public contributors participated in the first workshop. During the workshop we spent the first part of the session getting to know each other using an ice-breaker before talking about the purpose of the public involvement. The main focus of this first workshop was to ask for advice on how to go about recruitment for the *At Risk* study to ensure diversity. After the first workshop, public contributors were asked to reflect on the comic book idea and to come to the second workshop with some ideas of how to effectively develop the fictional characters as part of the comic book, or to suggest alternative visual elicitation tools, to help with clear and effective communication of bowel cancer risk. While the original idea of a comic book came from the research team, there was collective agreement that the comic book approach would be a useful research tool.

**Fig. 1:**
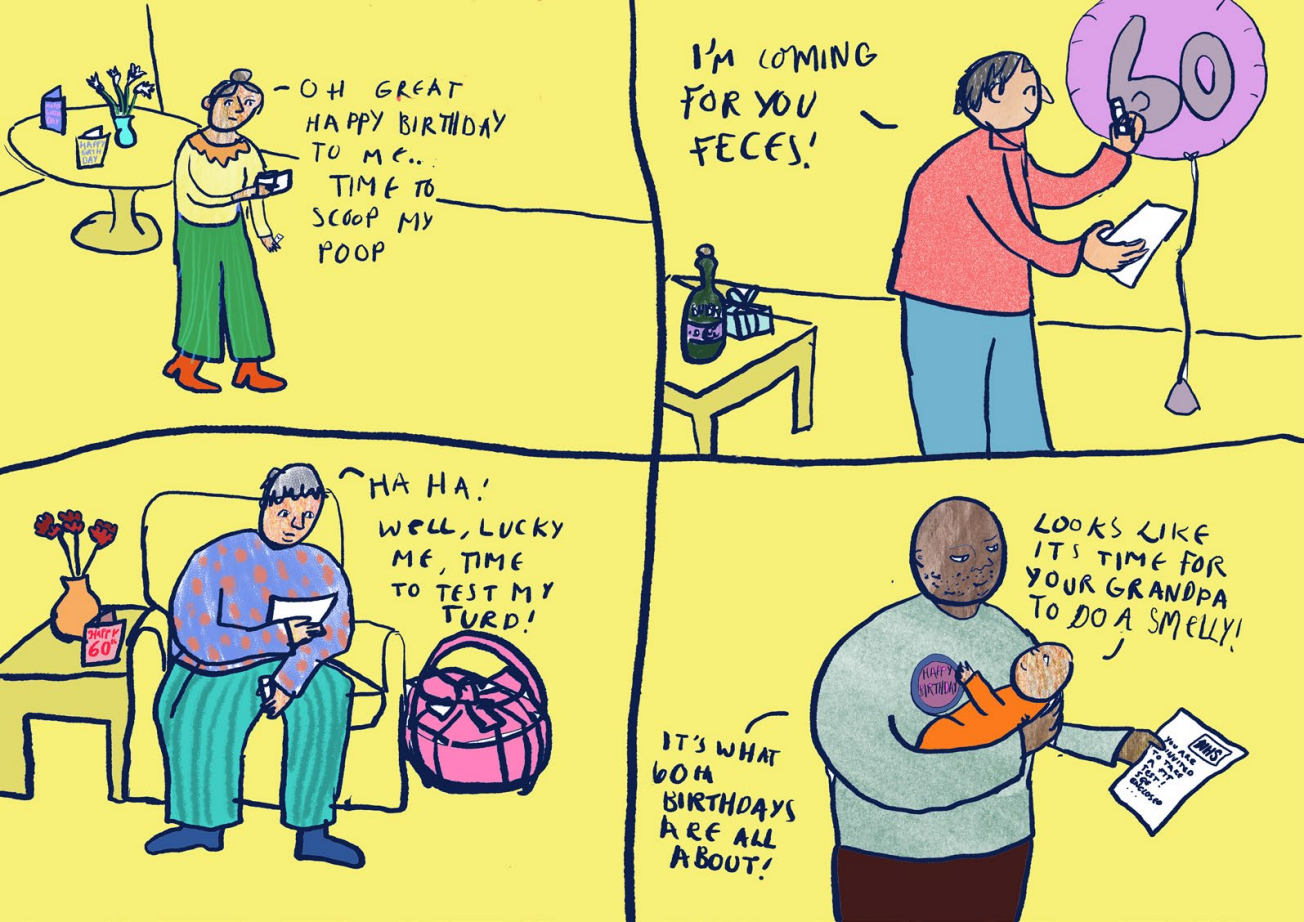
Panel style design

**Fig. 2:**
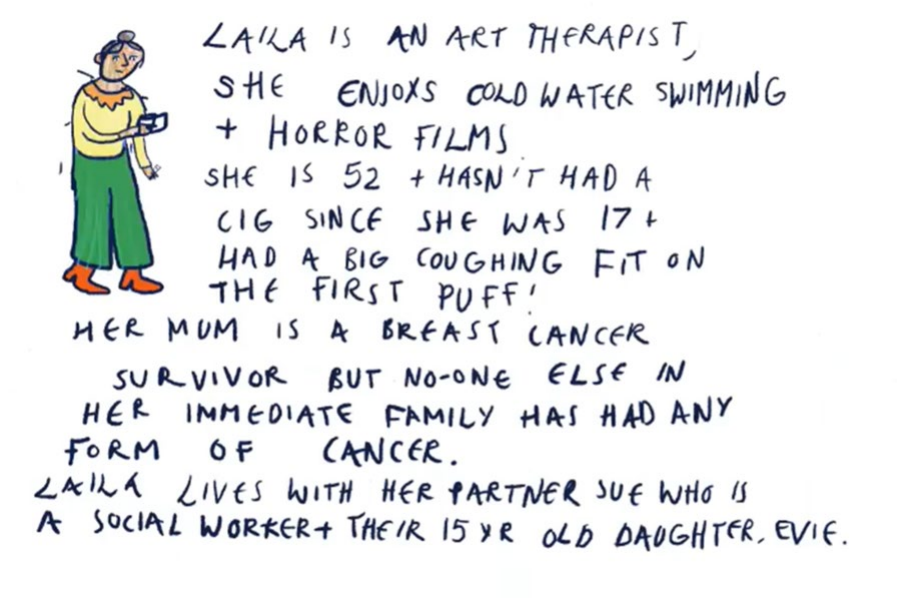
Narrative style design

**Fig. 3:**
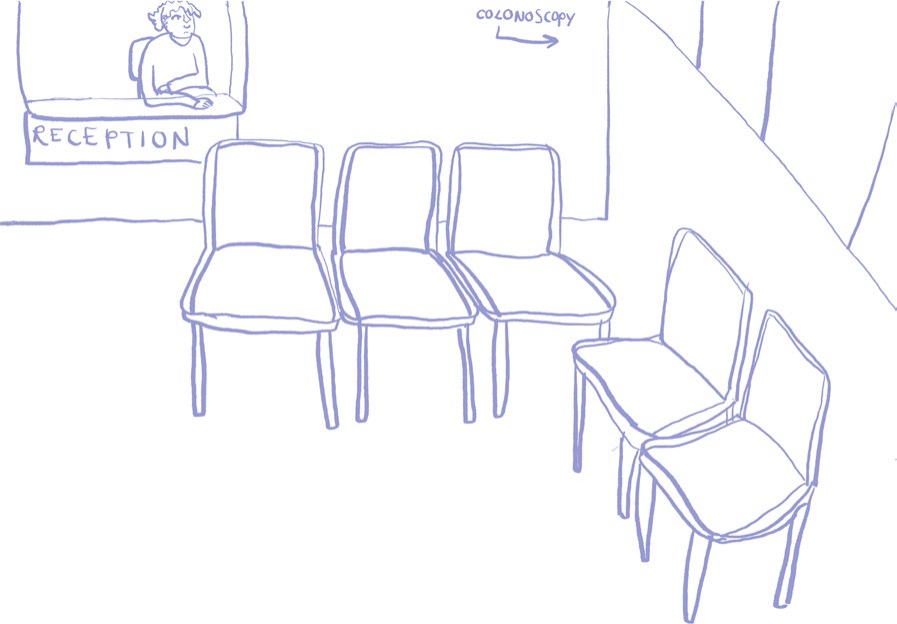
Clinical waiting room

**Fig. 4:**
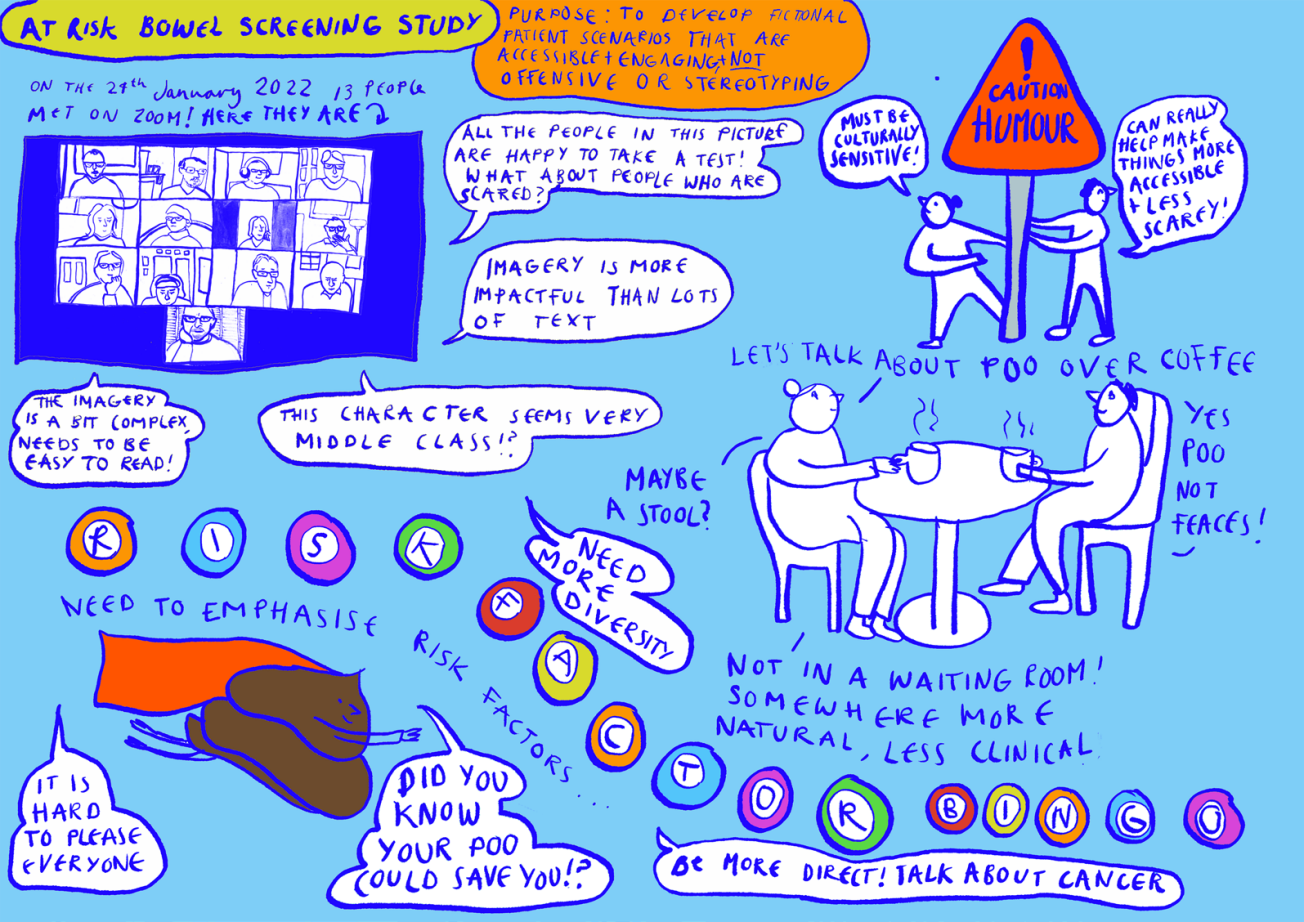
Visual notes

A local freelance cartoon artist, Lilly Williams, was commissioned for this project. She was briefed on the aims of the project and produced some initial sketches (Figs. 1, 2) for discussion in the second workshop. In preparation for the second workshop, she reflected on the discussion of the first workshop and created a blank waiting room scene (Fig. 3), which was used to make visual notes in real time as the workshop discussions progressed. Visual note-taking (Fig. 4) in the workshop helped to capture the discussions in real-time.

Nine out of ten public contributors who participated in the first workshop returned to the second workshop and the remaining public contributor contributed via email. The second workshop discussions centred around the following questions:

1. What do you think of the comic book approach—bringing together some characters with different levels of risk in a story?
  What do you like about this?
  Can you see any downsides?
  Can we get across the different risk factors clearly and consistently?
2. What do you think about using humour to make the topic seem less daunting?
  What do you like about this?
  Can you see any downsides?
  Will this appeal to most people, or put anyone off?
3. We have used a 60th birthday theme in the comic because most people in England receive their first home testing kit at 60. In Scotland and in other parts of the UK screening begins at a lower age.
  Should we remove this? Would it still make sense?
  Should we have different birthdays as well? Would that be confusing?
  Are there any other ways to show the age range of the screening programme visually?
4. Have we missed anything else important or essential?
5. Are there any other ways of creating fictional patients/communicating personal bowel cancer risk in a visual way?

After both of the workshops had taken place, the artist produced the final artwork and this was shared with the public contributors to ensure we had captured the essence of the discussions from the second workshop and they were given the opportunity to share their thoughts, with scope to make amendments. Public contributors were also invited to complete an online evaluation on Qualtrics, five of whom completed the evaluation. Some of the feedback from the evaluation is discussed in this article. We have also integrated some of the feedback we received about the comic book in the *At Risk* focus groups with members of the public and healthcare professionals to reflect different stakeholders perspectives.

## Benefits

**Fig. 5:**
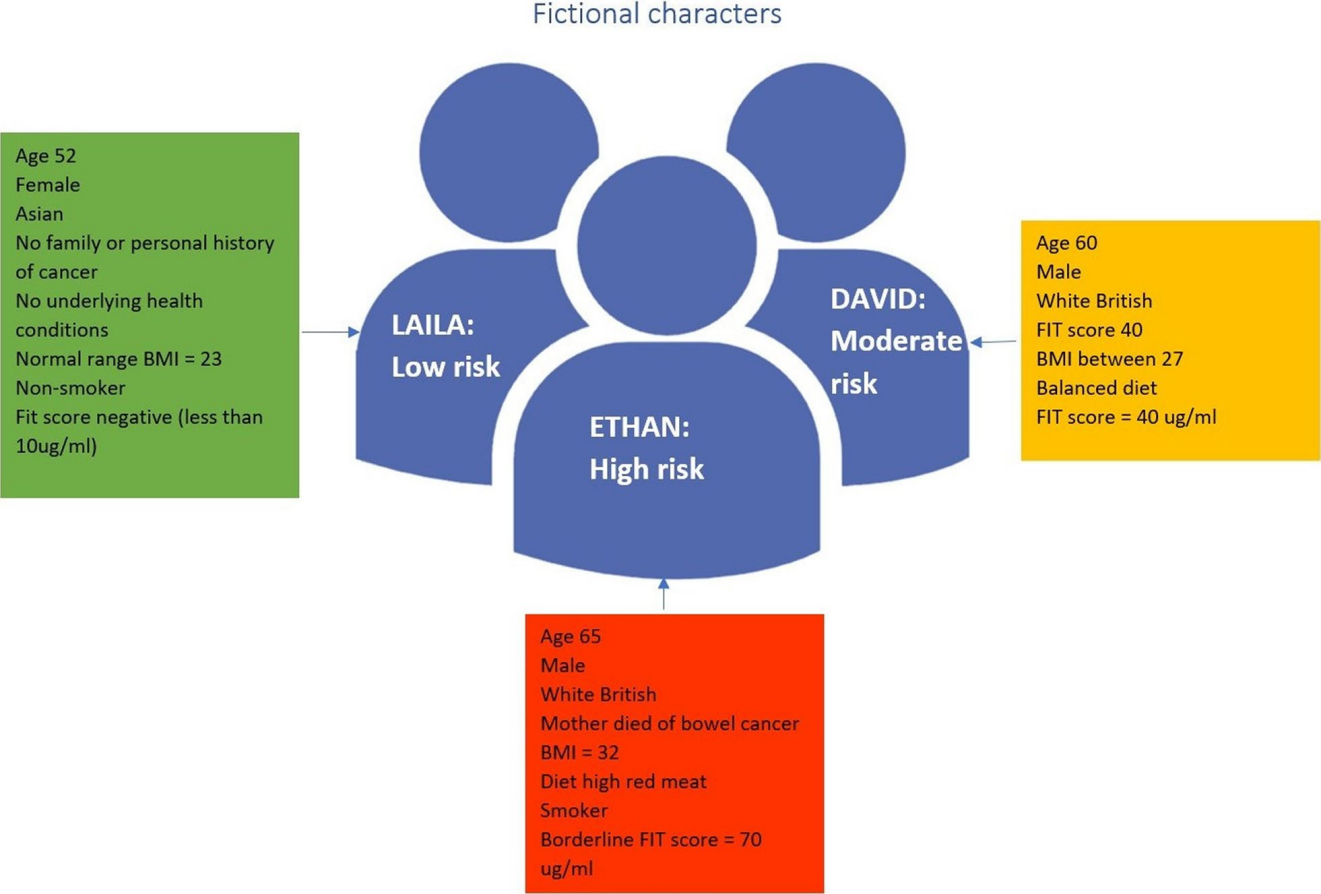
Blobs

One of the biggest benefits of working with diverse perspectives is the different skillsets and expertise, allowing for ideas to grow organically. This was a real strength of this comic book development and co-creation process, since the final artwork was very different from the fictional characters the research team started with. The original characters were referred to as ‘blobs’ (Fig. 5) by one of our public contributors, without personality and not particularly easy to relate to. This could have affected the way in which people responded to the characters if they were not easily relatable.

Initial comic sketches comprised three characters, one each assigned to low, moderate and high risk of bowel cancer based on various risk factors. These three characters were developed into six more personable and realistic characters (Joseph, Zahra, Mina, Jeff, Deborah and Paul), two illustrating each level of risk, which enabled us to tell a more convincing story. The final version of the comic book can be found here: https://www.yumpu.com/en/document/read/66455949/did-you-know-your-poo-could-save-you

It was reassuring to receive largely positive feedback about the accessibility of the comic book from the healthcare professionals during the *At Risk* focus groups, as captured in the following:

…it's just having clear messages and (…) by way of what you've developed there with that comic book, it helps to disseminate the message and more of a clear sort of very accessible way.

(General Practitioner, Yorkshire and Humberside, Focus Group).

The added value of the comic book approach was that discussions about sensitive health issues, such as bowel cancer risk and screening, were able to be approached in an indirect way, without drawing attention to an individual’s own level of risk, but still helped with personal identification of bowel cancer risk factors:

I think trying to put it into a simple language and how it can potentially affect them or make a story a little bit more real to them sometimes makes it a bit more personal.

(Clinical Programme Manager, London, Focus Group)

Furthermore, co-creation helped the researchers to think more creatively beyond the research context and consider other possible purposes for the comic book in addition to it being used as a research tool to generate conversation about a complex and sensitive topic and think about wider implementation in healthcare settings to inform screening decisions and even more broadly to raise awareness of screening and bowel cancer risk.

## Challenges

Despite the many benefits to co-creation, it can also present some challenges. One of the main challenges encountered related to how the researchers described the purpose of the comic book to those involved in co-creating the comic book. Most of the public contributors had some experience of public involvement before, to varying degrees, but not research. On reflection, we could have spent more time in the first workshop trying to explain how the tool would be used in the *At Risk* focus groups.

Another challenge was trying to reach a compromise when public contributors differed in their views on how to portray the fictional characters to illustrate different risk factors, as discussed under the comic book design issues below.

Additionally, despite a largely positive response from stakeholders (public contributors and research participants), comic books are not suited to everyone. Indeed, one of the research participants in the *At Risk* study would have preferred more traditional methods of receiving information about cancer risk to a comic book style:

…for me personally I would assimilate information much easier if it was not so much in comic book style.

(Research participant, Focus Group)

Other researchers have also identified similar barriers to the use of comic books in providing health information due to a lack of familiarity with educational comic books [7].

## Comic book decisions

During the co-creation process, some of the public contributors expressed reservations about the mock designs the artist had prepared for the comic book. These reservations could be grouped into the following design issues: language, style, characterisation, scenery and cultural sensitivity. Each will now be discussed in turn.

### Language

Comic book approaches may not always be recognised as comic books in the purest sense since they do not always conform to traditional conceptions of a comic book with respect to humour. Rainford [18] argues that the use of humour can potentially trivialise research and for this reason sometimes humour is avoided. In this project, we tried to have a careful balance of humour to ensure we were not being offensive or trivialising bowel cancer in any way. It was also recognised that humour is subjective and not everyone will share the same sense of humour. As a result, we wanted to be culturally sensitive without losing the lighthearted and accessible artistic style of the cartoonist. We therefore spent some time discussing the best terms to use for key elements of our story. Some of the public contributors were not keen on the use of different terms to describe poo (see Fig. 4). One participant said “call poo, poo, not faeces, stool or turd!” Others said that turd might be regarded as offensive to some people. We therefore used the term poo throughout the comic book and in the title, ‘Did you know your poo could save you?’, as suggested by one of the public contributors.

Some of the lifestyle factors that contribute to bowel cancer risk, such as smoking, can be potentially stigmatising and leave people feeling judged for their behaviour, as has been found in previous research [19]. The artist took this into consideration and created a scene between a mother and son in which the risk of smoking is discussed with sardonic humour in the context of a loving relationship. There were also sensitivities about how best to present weight as a risk factor. The artist developed a character who was overweight, Mina, but only at moderate risk of bowel cancer, and presented her openly discussing her weight in a conversation where she encouraged her husband, Jeff, to take part in bowel screening. We ended up using a mixture of terms to describe weight.

Overall, even though there were some language challenges, the narrative came together after we were able to iron out some initial concerns and this is reflected in the following feedback from one of the public contributors:

I liked the narrative of the comic book.

(Public contributor, Scotland, evaluation form)

### Style

**Fig. 6:**
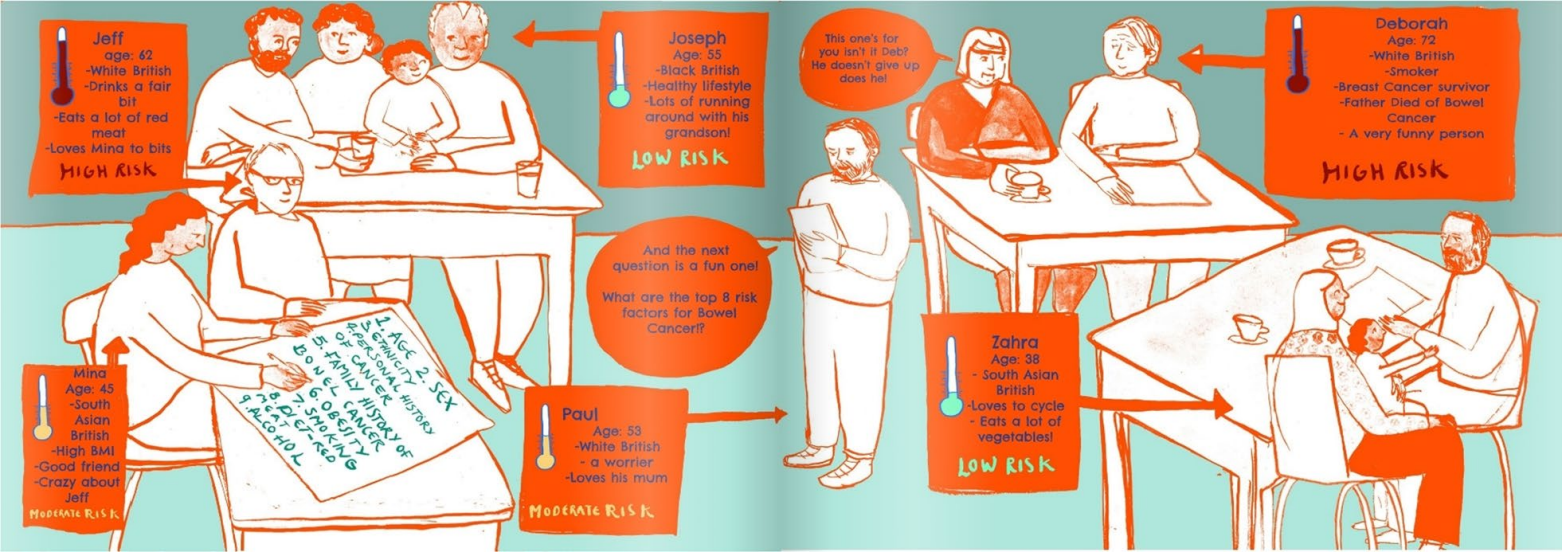
Community quiz

There was an interesting debate between public contributors as to whether the characters should be standardised (e.g. listing risk factors for comparability and presented as faceless ‘screen beans’) or developed into individuals with a personal story. The researchers and artist tried to reach a compromise by having some comparability between characters in the final community quiz scene (Fig. 6) but we had to make a choice about whether to choose a narrative style or a more standardised one. We opted for building a story around unique characters who were part of the same community. We wanted to make sure that the characters did not become stereotypes, detached from real life and harder to relate to. We also decided that a narrative style was better suited to a comic book format. Some of the public contributors felt strongly that the narrative approach was not the right approach and detracted from the message we were trying to convey. This point of view is represented in the visual notes (Fig. 4) in the speech bubble stating that “Imagery is more impactful than lots of text”. Therefore, narration was kept to a minimum and interspersed with lots of imagery, opting for a more hybrid style.

### Characterisation

The artist’s early ideas for the comic book included a set of panels giving pen portraits of each fictional character. The research team strived for diversity, placing a particular focus on ethnicity, but what stood out to the public contributors was a lack of class diversity. For instance, one of the original characters, Laila, came across as a middle-class stereotype given her occupation and hobbies (see Fig. 2). Therefore, it was decided not to include these pen portraits in the final version of the comic book, instead placing our focus on the risk factors associated with bowel cancer. However, we did include some personal characteristics in the final community quiz scene where all the characters appear together with their individual risk level (Fig. 6). This served a dual purpose, to make the characters more relatable and to bring in humour alongside the list of risk factors. This was also referred to by one of the healthcare professionals who took part in one of the *At Risk* focus groups:

…one of the things that I like about that are about your comic strip in the idea that talking around the table.

(Gastroenterologist, Yorkshire and Humberside, Focus Group)

### Scenery

**Fig 7:**
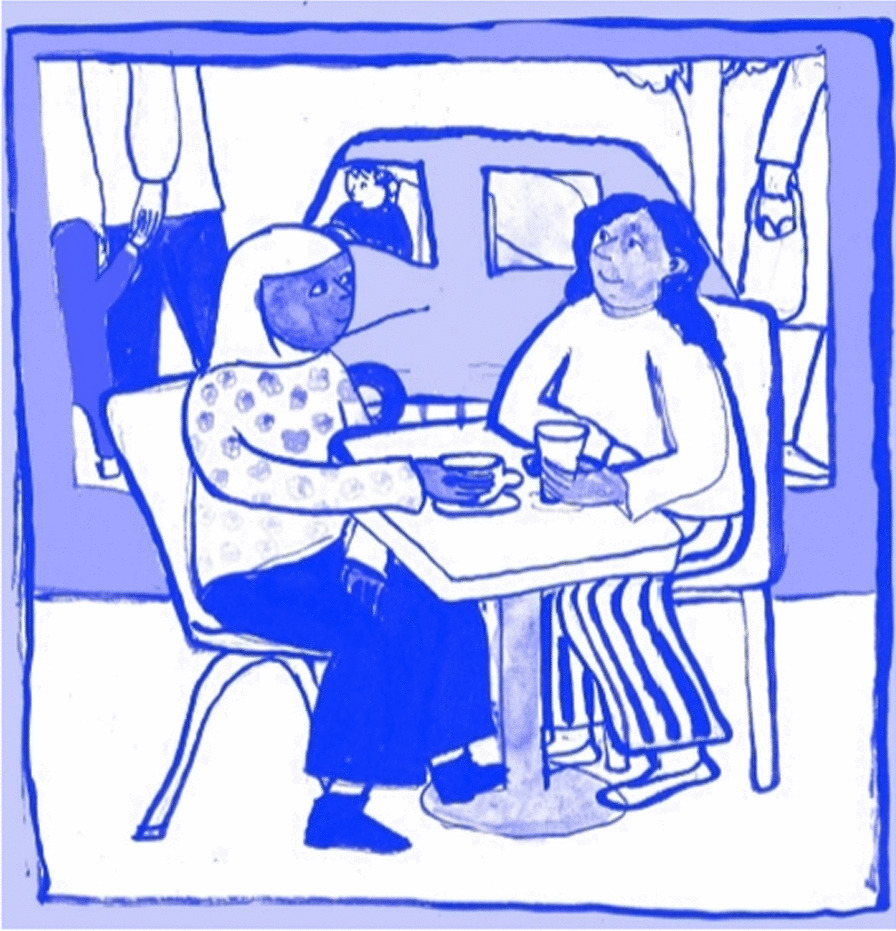
Tea scene

There was consensus amongst the public contributors that they were not keen on the waiting room setting being used in the comic book. Instead, they wanted the characters to meet somewhere more natural and less clinical, somewhere sociable like a café. The universality of tea was mentioned by a public contributor, which we incorporated into one of the scenes (Fig. 7). This reflects the fact that bowel screening is initially completed at home using a home testing kit and the clinical setting would only feature if someone received a positive test result and was called for a colonoscopy.

There was also discussion about the characters all originally being perceived as positive about participating in bowel cancer screening, but this did not reflect the inequalities in uptake and anxieties that some people may experience and might act as a barrier to completing a FIT test. We therefore took this on board with the first character, Joseph, who was less than enthusiastic about ‘scooping his poop’ but with the encouragement of his grandson completes it.

Some of the public contributors highlighted that bowel cancer screening is not necessarily a priority to everyone and, as one of them aptly voiced: “people have more to look forward to on their 60th birthday than sending off a FIT test”. We did decide to keep one scene containing an invitation to bowel cancer screening which arrived on Joseph’s birthday, however we did not extend this to the other characters.

### Cultural sensitivity

Cultural sensitivity came through strongly in our workshop discussions and we followed this up by presenting the comic book at a meeting of the Humber All Nations Alliance network. This meeting included representatives from local Black, central European and Chinese community organisations. We asked if the message of the comic book was conveyed clearly, if it was presented in a sensitive way, and if it caused offence for any reason. The group were overwhelmingly positive about the comic book. They shared important suggestions and ideas about how the comic book could be developed (e.g. as a live performance or translated into languages other than English), and how it could be used for other purposes (e.g. to get over the stigma of talking about bodily functions and cancer risk in different communities, and increase cancer awareness). We were open to changing the comic book if any cultural sensitivities were breached, but the group did not think this was needed.

## Lessons learned

It can be tricky managing different perspectives and seem impossible to reconcile everyone’s views. At times, the research team and artist went with the majority view and, other times, we had to make a difficult decision where there was no clear consensus. However, public contributors were fully informed about what was decided and we shared the final version of the comic book with them and we also asked them to complete an evaluation form. This meant that everyone felt heard and included, and could see that their opinions had influenced the project, as described by one of the contributors in the evaluation:

Lilly [the artist] has taken onboard the differing opinions of the group to produce a comic that reflects everything we said.

(Public Contributor, England, evaluation form)

One notable limitation of our project study was that the research team started with a fixed idea of what the comic book would be used for. This meant that we were not open to considering alternative uses for the comic book until after the artwork had already been designed. Several individuals who were involved in both the public involvement workshops and focus groups with members of the public and healthcare professionals supported the idea of using the comic book as a health education tool. The team has since reflected on this and agreed it would be worthwhile repurposing the comic book to promote awareness of bowel cancer risk and bowel cancer screening to the wider public. It is thought that the comic book could be adapted into a narrative or gist-based leaflet [20, 21], which have successfully been used to inform bowel cancer screening intention.

In addition, the team is also exploring other participatory arts-based approaches by partnering with local community arts organisations to turn the comic book into a theatrical performance and taking this to local communities that have lower bowel cancer screening uptake and longstanding health inequalities in the hope that it may encourage uptake, or at least informed decision-making about bowel cancer screening.

## Conclusion

The aim of the co-created comic book was to convey different levels of bowel cancer risk in a way that was accessible and engaging to members of the public who are of bowel cancer screening age. The comic book was used as a research tool in the *At Risk* focus groups to help to stimulate discussion about bowel cancer risk and risk-based screening in a sensitive and empathetic way.

Overall, the comic book was well-received in the focus groups by both members of the public and healthcare professionals, but it is important to note that comic books do not suit everyone and humour is not universal and so it is essential to carefully consider these aspects when considering whether it is an appropriate tool to use. Meaningful public is necessary to guide this decision.

In this article we have reflected on the process of co-creation, including the benefits, challenges and lessons learned, which may assist other health researchers who are considering adopting a similar approach. We recommend the following key ingredients for successful co-creation: (1) having a clear purpose and shared objective; (2) reaching an agreement on key design issues such as language, style and characterisation; and (3) being culturally sensitive.

Certainly, co-creation does not come without challenges but it is highly worthwhile and we would strongly encourage other researchers working in the field of cancer research to see the value in what can be achieved through creative arts-based health research. It not only helps to make research more creative and innovative but it also makes research more accessible and engaging. Moreover, it was recognised by healthcare professionals as an effective medium for communication about complex issues such as cancer risk.

## Data Availability

Anonymised transcripts are available on request.
